# Supplementary material for: Nanobodies against Plasmodium adhesins that block receptor engagement and malaria parasite invasion
Source: Biochem J. 2026 Jul 9;483(8):1473–88. doi: 10.1042/BCJ20260354 (PMC13358728; doi:10.1042/BCJ20260354)
Supplement: Supplementary Figures S1-S8 and Table S1 [file BCJ-2026-0354_supp.pdf]

Figure S1

A.

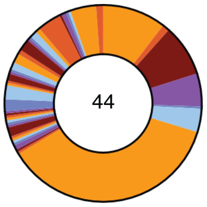

B.

Competing nanobody (columns); **Immobilised nanobody** (rows)

Immobilised nanobody	2886	2888	2891	2892	2893	2896	2897	2898	2905	2908	2909	2890	2900	2906	2887	2889	2894	2899	2901	2902	2903	2907	2895	2904	Group
2886	0	2	1	1	5	1	3	1	1	5	6	3	3	106	89	86	85	90	88	92	84	91	108	105	Group A
2888	3	7	4	2	7	3	5	2	1	6	5	4	3	101	92	85	85	88	87	92	83	89	110	105	Group A
2891	2	5	3	2	8	3	9	2	1	5	5	4	4	101	90	85	86	88	86	91	81	89	107	104	Group A
2892	2	2	3	2	6	2	3	1	2	4	5	3	3	100	87	85	81	86	86	91	81	88	107	104	Group A
2893	1	2	2	1	4	-0	2	0	2	2	5	2	2	94	91	88	87	91	92	96	88	89	110	108	Group A
2896	2	2	2	2	4	1	3	1	0	3	4	2	-0	88	89	87	84	86	90	94	84	91	110	106	Group A
2897	2	5	3	1	5	2	4	1	2	3	5	3	1	94	90	84	83	83	86	92	82	86	108	104	Group A
2898	1	5	3	1	7	4	13	-0	1	4	6	3	3	105	92	89	89	91	90	94	86	92	106	103	Group A
2905	1	5	4	1	8	3	8	2	1	4	6	3	3	105	91	86	87	86	87	92	83	88	107	105	Group A
2908	-0	0	-0	1	6	-1	1	-0	0	2	4	1	-1	87	85	83	85	86	88	93	84	91	109	107	Group A
2909	0	1	2	3	7	0	2	1	1	6	5	2	1	96	85	83	82	82	85	91	82	87	107	105	Group A
2890	1	4	3	1	6	2	4	2	2	4	5	3	1	78	89	84	82	86	86	91	82	88	106	103	Group B
2900	1	2	2	2	5	1	3	2	2	3	6	2	2	62	90	87	84	90	90	94	87	87	109	106	Group B
2906	79	83	76	66	94	56	72	112	53	46	82	21	13	7	90	89	84	86	88	94	84	90	113	107	Group C
2887	109	105	108	109	110	102	105	129	101	109	103	113	111	118	1	3	1	1	2	0	-0	3	127	116	Group D
2889	108	104	106	108	110	100	102	124	99	109	103	111	107	114	1	1	1	-1	1	1	1	1	128	117	Group D
2894	108	105	108	108	109	104	107	122	102	107	104	111	109	115	1	3	1	1	2	1	-0	2	123	115	Group D
2899	109	106	105	109	111	101	103	126	101	107	103	111	109	116	3	4	3	3	2	2	1	2	123	117	Group D
2901	108	104	107	108	110	103	105	126	100	106	101	112	109	114	1	3	1	1	1	0	-1	2	127	117	Group D
2902	109	111	107	109	113	101	103	129	101	108	105	112	111	116	6	3	4	3	2	4	4	3	129	118	Group D
2903	109	106	107	110	111	103	105	123	102	109	105	111	108	115	2	2	1	-0	2	2	1	1	122	115	Group D
2907	105	104	104	103	111	93	99	119	97	105	100	109	103	116	1	1	1	1	2	0	3	3	123	117	Group D
2895	105	110	104	108	108	95	99	121	98	103	100	108	105	114	124	118	122	113	117	127	111	120	3	3	Group E
2904	103	107	104	106	108	97	100	123	98	103	100	108	103	113	102	98	95	101	102	106	95	104	2	2	Group E

C.

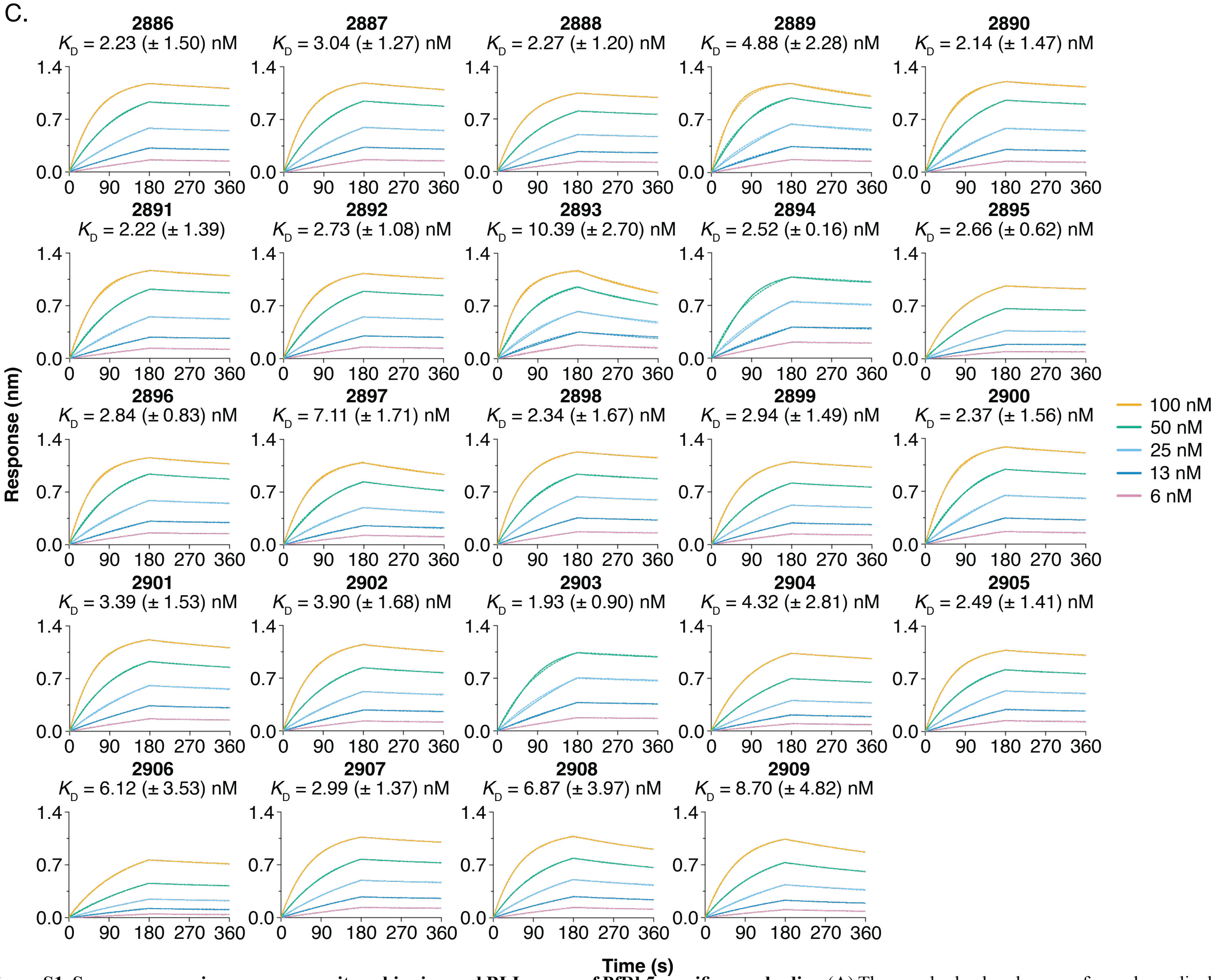

Figure S1. Sanger sequencing summary, epitope binning and BLI curves of PfRh5 specific nanobodies. (A) The nanobody clonal groups from phage display panning against PfRh5 identified using Sanger sequencing. The number in the inner circle indicates the number of distinct clonal groups based on the CDR3 sequences. Colored pie slices are proportional to the number of clonally related sequences. (B) Epitope binning competition experiments using BLI with PfRh5. Immobilized nanobodies are indicated on the left column and nanobodies pre-incubated with PfRh5 at a 10:1 molar ratio are indicated on the top row. Binding of PfRh5 pre-mixed with nanobody was calculated relative to PfRh5 binding alone, which was assigned to 100%. The green and white boxes represent non-competing and competing nanobodies, respectively. (C) BLI affinity measurements using a two-fold dilution series of PfRh5 from 6 – 100 nM binding to immobilized PfRh5 nanobodies. Measurements were plotted (solid line) and fitted to a 1:1 binding model (dashed line). Mean K_D values with standard deviations are indicated, and representative binding curves are shown from two independent experiments.

Figure S2

A.

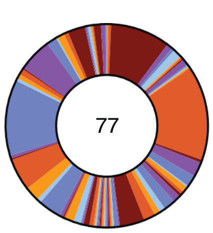

B.

Competing nanobody

Immobilised nanobody	2858	2860	2862	2863	2864	2865	2867	2869	2871	2872	2875	2877	2859	2861	2868	2870	2873	2874	2866	2876	Group
2858	2	3	0	-2	2	2	1	1	3	4	4	3	118	111	109	110	118	110	108	115	Group A
2860	4	4	5	3	4	5	4	6	6	8	8	8	116	110	113	110	116	113	109	116	
2862	3	3	3	3	3	4	1	5	4	6	6	6	116	110	114	112	118	115	108	112	
2863	3	3	3	1	3	3	2	3	4	6	5	5	115	109	110	108	115	110	105	112	
2864	3	3	3	0	3	3	2	2	3	5	5	4	115	110	107	106	117	107	105	113	
2865	3	3	0	-2	3	2	1	1	4	5	2	3	115	106	102	103	110	99	107	112	
2867	3	4	4	4	4	5	2	7	7	8	8	8	131	122	128	129	130	129	109	130	
2869	2	2	3	3	1	3	2	4	4	5	4	3	118	110	112	112	111	110	110	115	
2871	5	4	3	2	4	5	3	3	5	6	5	6	118	109	110	111	114	110	108	116	
2872	2	2	-0	-2	1	1	1	1	1	4	2	4	124	117	109	119	121	113	112	118	
2875	3	0	3	0	2	2	1	3	2	5	1	5	116	111	111	111	113	108	107	112	
2877	3	2	3	1	2	3	2	4	3	7	4	4	110	105	108	108	110	106	107	113	
2859	112	77	119	94	111	118	92	82	106	100	95	87	1	4	4	2	6	15	104	118	Group B
2861	97	59	116	80	95	99	85	71	89	102	77	77	5	6	8	3	9	17	102	119	
2868	91	89	124	94	97	91	105	90	105	98	85	95	6	5	9	5	3	16	106	121	
2870	96	78	127	88	82	90	93	87	103	109	73	88	7	3	10	3	3	18	105	116	
2873	113	77	141	104	121	114	108	89	117	118	86	103	3	4	3	-1	0	8	109	126	
2874	93	89	171	99	93	91	158	122	126	123	96	124	16	9	14	6	6	22	119	151	
2866	104	102	103	103	109	108	107	103	103	100	105	104	115	111	109	110	114	109	-0	3	Group C
2876	112	113	91	115	120	121	113	117	114	115	129	113	125	114	116	113	117	113	3	2	

C.

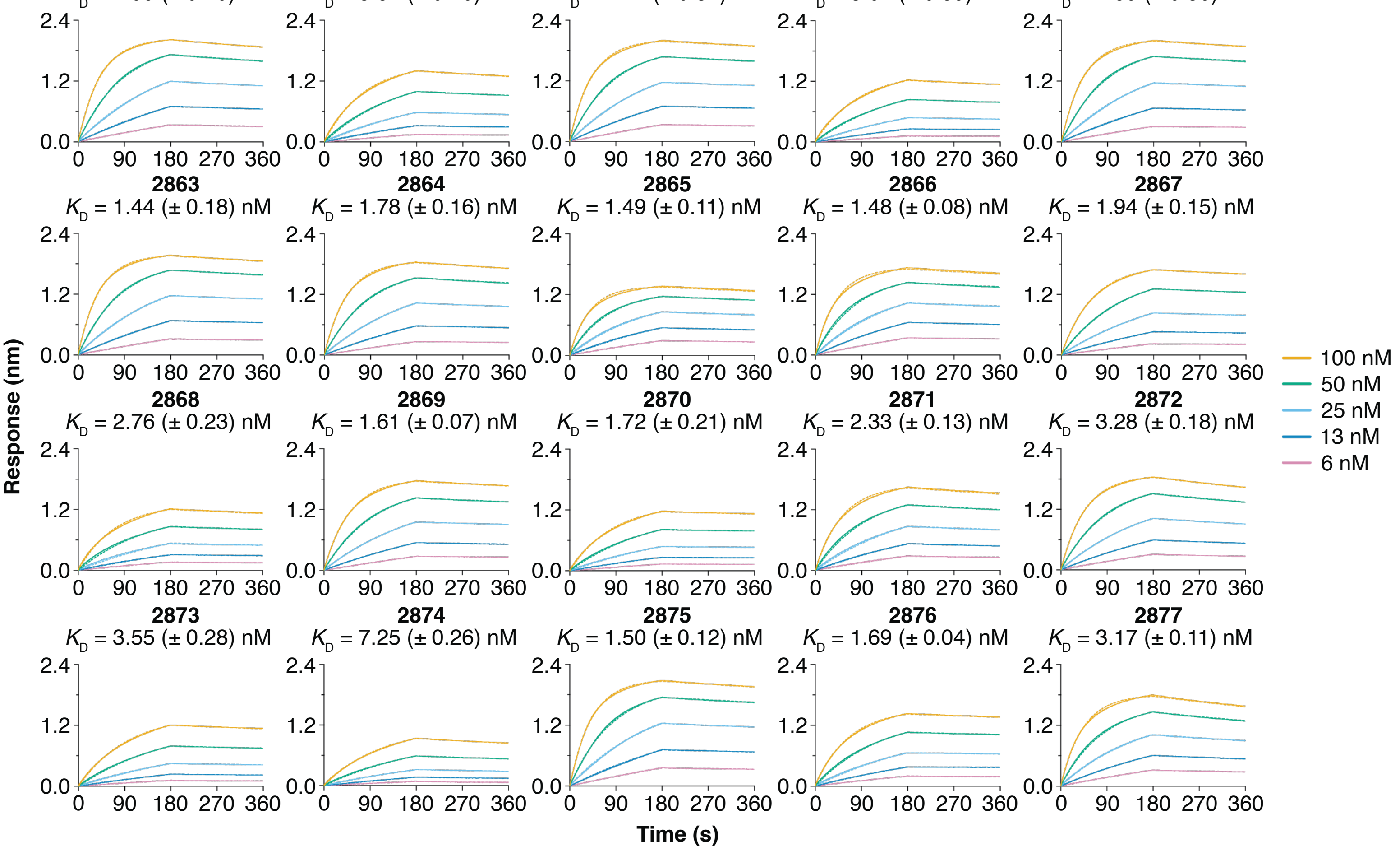

Figure S2. Sanger sequencing summary, epitope binning and BLI cruves of PfRh4 specific nanobodies. (A) The nanobody clonal groups from phage display panning against PfRh4 identified using Sanger sequencing. The number in the inner circle indicates the number of distinct clonal groups based on the CDR3 sequences. Colored pie slices are proportional to the number of clonally related sequences. (B) Epitope binning experiments using BLI with PfRh4. Immobilized nanobodies are indicated on the left column and nanobodies pre-incubated with PfRh4 at a 10:1 molar ratio are indicated on the top row. Binding of PfRh4 pre-mixed with nanobody was calculated relative to antigen binding alone, which was assigned to 100%. The green and white boxes represent non-competing and competing nanobodies, respectively. (C) BLI affinity measurements using a two-fold dilution series of PfRh4 from 6 – 100 nM binding to immobilized PfRh4 nanobodies. Measurements were plotted (solid line) and fitted to a 1:1 binding model (dashed line). Mean K_D values with standard deviations are indicated, and representative binding curves are shown from two independent experiments.

Figure S3

A.

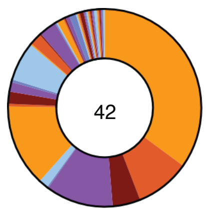

B.

Competing nanobody (columns) / **Immobilised nanobody** (rows)

Immobilised nanobody	2910	2913	2914	2919	2920	2922	2924	2925	2931	2934	2936	2937	2923	2917	2929	2930	2933	2916	2932	2915	2911	2927	2938	
2910	2	2	2	2	2	2	2	2	2	5	1	123	129	126	125	120	136	126	121	123	120	129	123	Group A
2913	3	2	2	2	2	2	3	3	3	7	2	126	130	127	126	122	136	125	123	124	124	130	126	
2914	1	1	1	2	2	1	2	2	2	4	1	123	129	126	124	120	135	124	120	121	121	128	125	
2919	3	2	2	2	3	2	3	3	3	6	2	124	129	128	126	121	136	128	124	125	124	130	126	
2920	1	1	1	1	0	1	1	0	0	3	1	123	128	126	124	120	134	125	120	121	121	129	121	
2922	4	2	3	3	4	2	4	4	3	7	3	126	131	128	127	123	138	129	124	123	125	129	129	
2924	2	2	2	3	2	2	3	3	2	6	2	124	129	127	126	122	136	127	123	122	122	131	97	
2925	2	2	2	2	2	2	3	2	2	5	2	125	130	127	127	123	136	127	121	123	123	129	125	
2931	3	3	3	3	2	2	3	3	3	5	2	127	131	128	127	124	136	128	121	123	123	129	125	
2934	3	3	3	3	4	3	4	3	4	5	3	126	130	127	130	123	137	132	122	124	124	131	128	
2936	1	1	1	1	1	1	1	0	1	4	1	126	130	129	127	122	135	127	122	122	123	131	125	
2937	129	130	130	130	128	130	129	128	133	127	131	17	4	5	4	4	4	3	5	72	87	59	126	Group B
2923	132	129	131	131	133	131	132	132	135	131	134	59	2	8	3	4	2	2	3	123	125	128	125	
2917	129	130	130	131	131	131	130	129	133	130	133	52	2	7	1	2	2	1	1	110	115	109	127	
2929	134	134	135	133	137	135	135	135	136	133	136	72	3	12	2	5	3	2	5	130	130	134	131	
2930	134	133	134	132	135	134	135	134	136	132	136	67	3	11	3	5	3	4	5	128	128	130	131	
2933	129	128	129	129	132	131	130	129	131	128	134	49	2	7	2	3	2	2	1	123	123	127	126	
2916	131	130	131	131	133	133	132	130	135	133	134	77	2	12	2	5	3	3	4	104	125	129	127	– Group C
2932	135	135	137	133	137	135	137	136	136	134	136	76	2	13	2	4	2	3	2	70	11	25	133	– Group D
2915	131	129	128	129	133	118	132	131	134	131	136	117	118	121	124	120	135	21	5	7	3	3	127	– Group E
2911	131	130	129	130	133	131	130	130	133	129	135	126	135	131	129	126	138	119	33	7	2	2	127	– Group F
2927	133	133	133	133	136	132	134	132	134	131	138	118	132	128	130	125	139	121	39	10	4	3	5	– Group G
2938	130	131	133	131	134	133	100	131	133	132	132	128	131	130	130	126	137	130	125	127	128	132	2	– Group H

C.

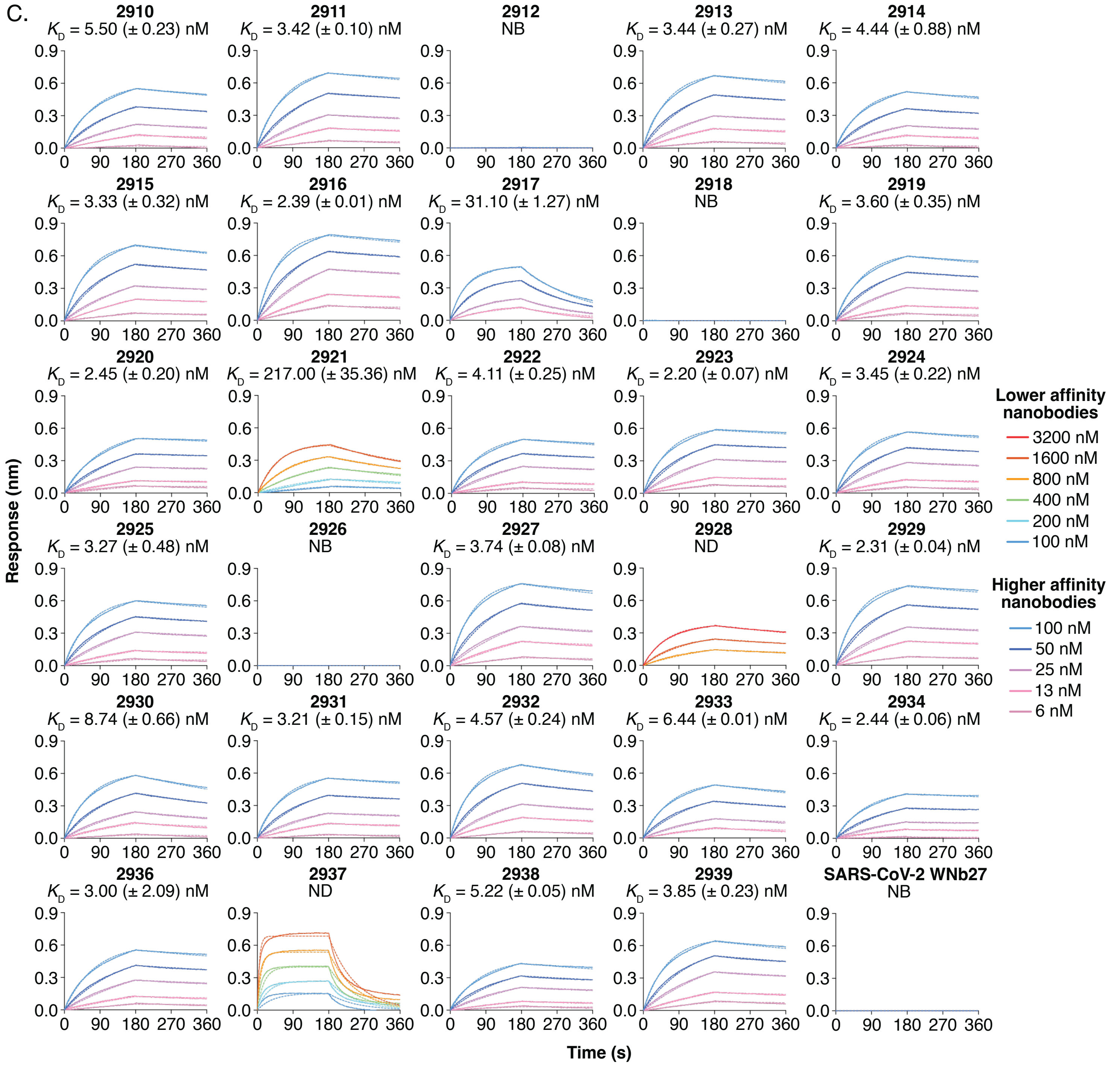

Figure S3. Sanger sequencing summary, epitope binning and BLI curves of PvRBP2b specific nanobodies. (A) The nanobody clonal groups from phage display panning against PvRBP2b identified using Sanger sequencing. The number in the inner circle indicates the number of distinct clonal groups based on the CDR3 sequences. Colored pie slices are proportional to the number of clonally related sequences. (B) Epitope binning experiments using BLI with PvRBP2b. Immobilized nanobodies are indicated on the left column and nanobodies pre-incubated with PvRBP2b at a 10:1 molar ratio are indicated on the top row. Binding of antigen pre-mixed with nanobody was calculated relative to antigen binding alone, which was assigned to 100%. The green and white boxes represent non-competing and competing nanobodies, respectively. (C) BLI affinity measurements using a two-fold dilution series of PvRBP2b from 100 – 3200 nM for lower affinity nanobodies and 6 – 100 nM for higher affinity nanobodies. Measurements were plotted (solid line) and fitted to a 1:1 binding model (dashed line). Mean K_D values with standard deviations are indicated, and representative binding curves are shown from two independent experiments. NB, no binding; ND, not determined due to poor fitting of the measured data to the binding model.

Figure S4

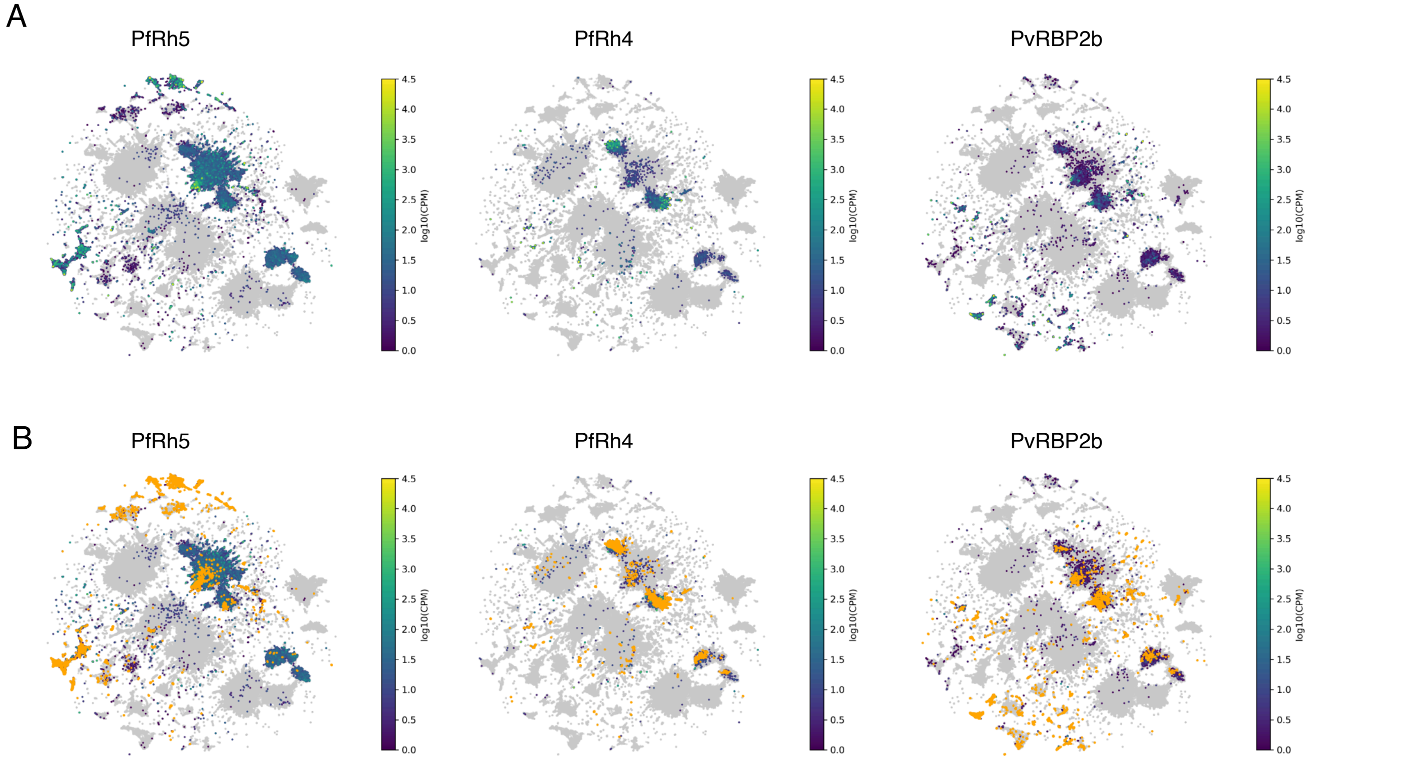

Figure S4. Selection hotspots within the sequence space of observed nanobody variation. (A) Each point represents a unique nanobody sequence that has been embedded into a vector using the AntiBERTy antibody language model and reduced into 2D space for visualisation via UMAP. Grey represents all observed nanobody variation prior to phage display selection. Nanobody sequences remaining after the second round of phage display selection against PfRh5, PfRh4 and PvRBP2b respectively are coloured according to their abundance (log10 of CPM). (B) Sequences highlighted in orange are from the same clonal group as nanobodies selected for characterisation in this study against PfRh5, PfRh4 and PvRBP2b.

Figure S5

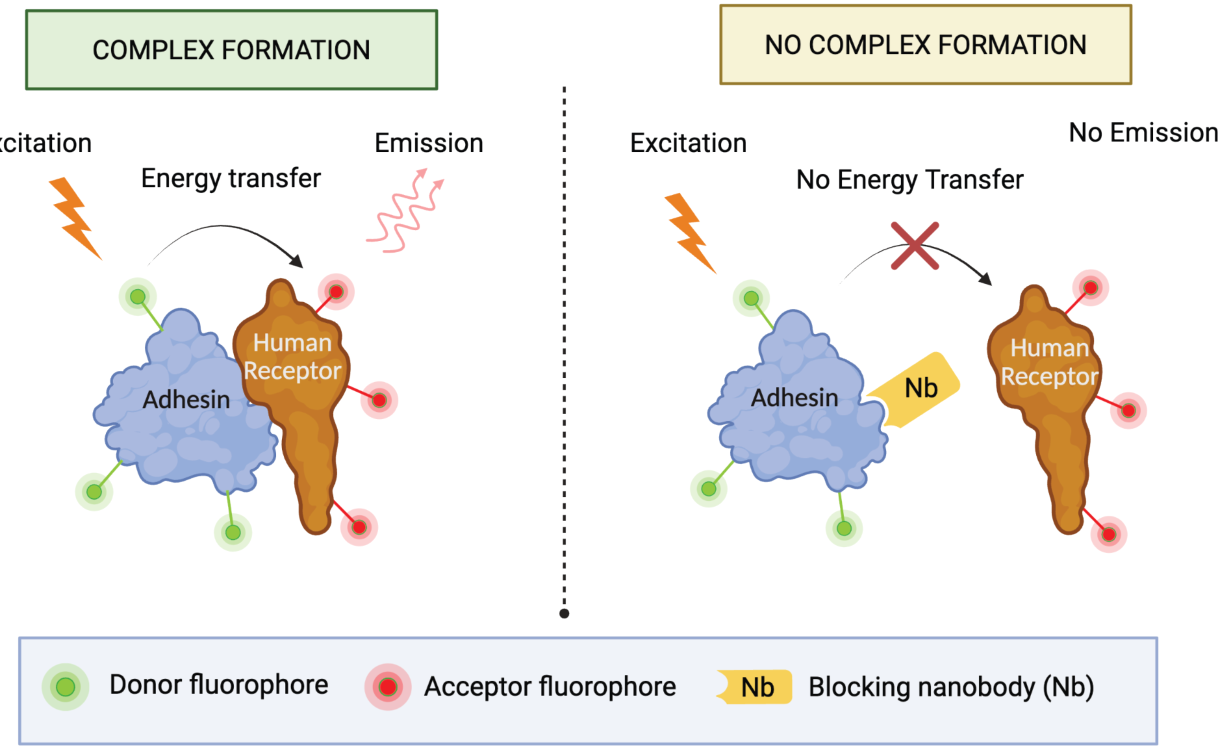

Figure S5. Schematic of FRET experiment. The malaria parasite adhesin is labelled with donor fluorophore and the human receptor is labelled with acceptor fluorophore. When the parasite adhesin and receptor interact and form a complex, the fluorophores are brought into close proximity. Upon donor fluorophore excitation, the close proximity enables energy transfer (FRET) from the donor to acceptor fluorophore, resulting in acceptor fluorophore emission. Nanobodies that block adhesin and receptor complex formation reduces FRET and acceptor fluorophore emission. Created in https://BioRender.com

Figure S6

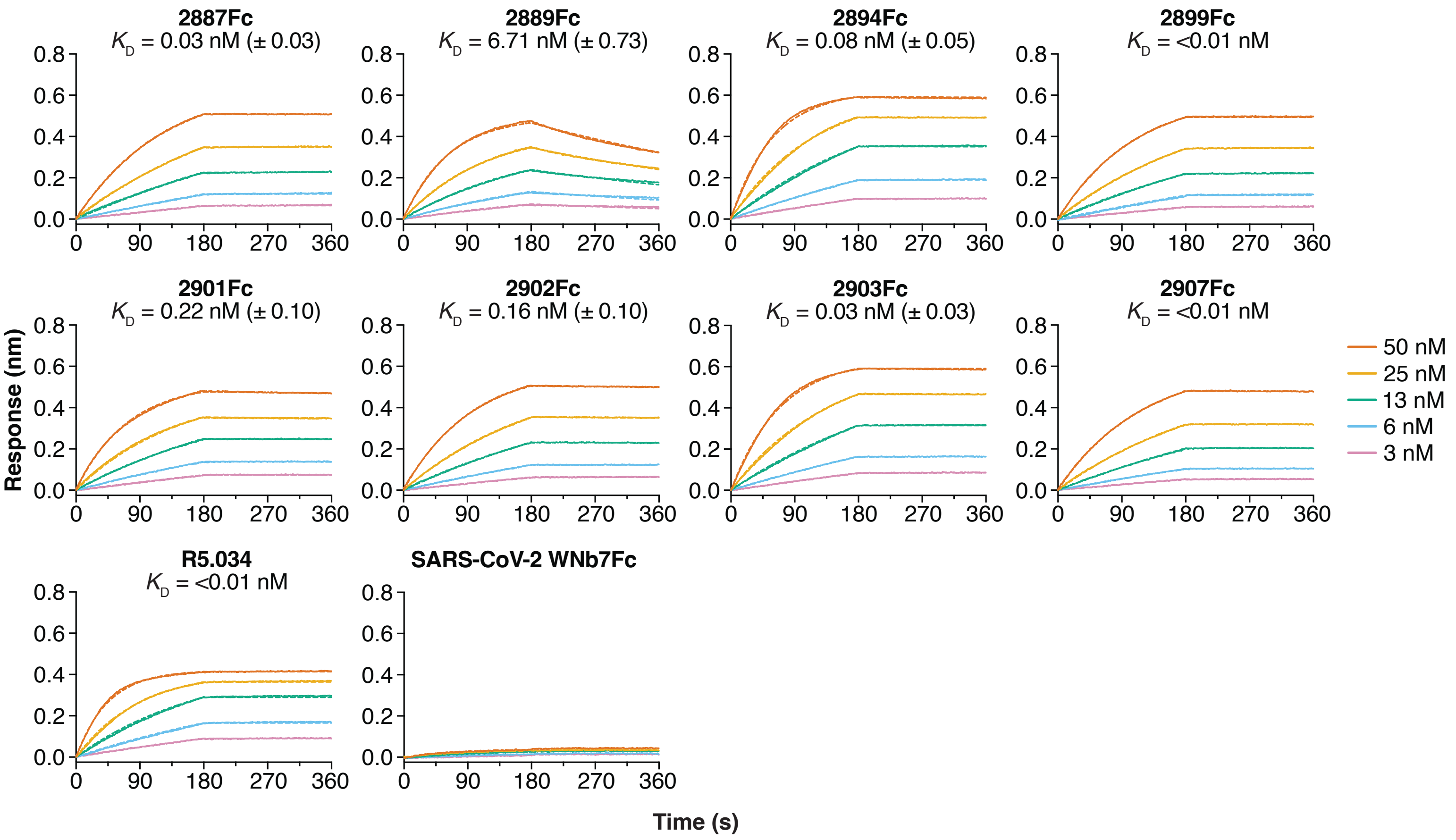

Figure S6. BLI affinity curves for PfRh5 nanobody-Fcs. BLI affinity measurements using a two-fold dilution series of PfRh5 from 3 – 50 nM binding to immobilized PfRh5 nanobody-Fcs. Measurements were plotted (solid line) and fitted to a 1:1 binding model (dashed line). Mean K_D values with standard deviations are indicated, and representative binding curves are shown from two independent experiments.

Figure S7

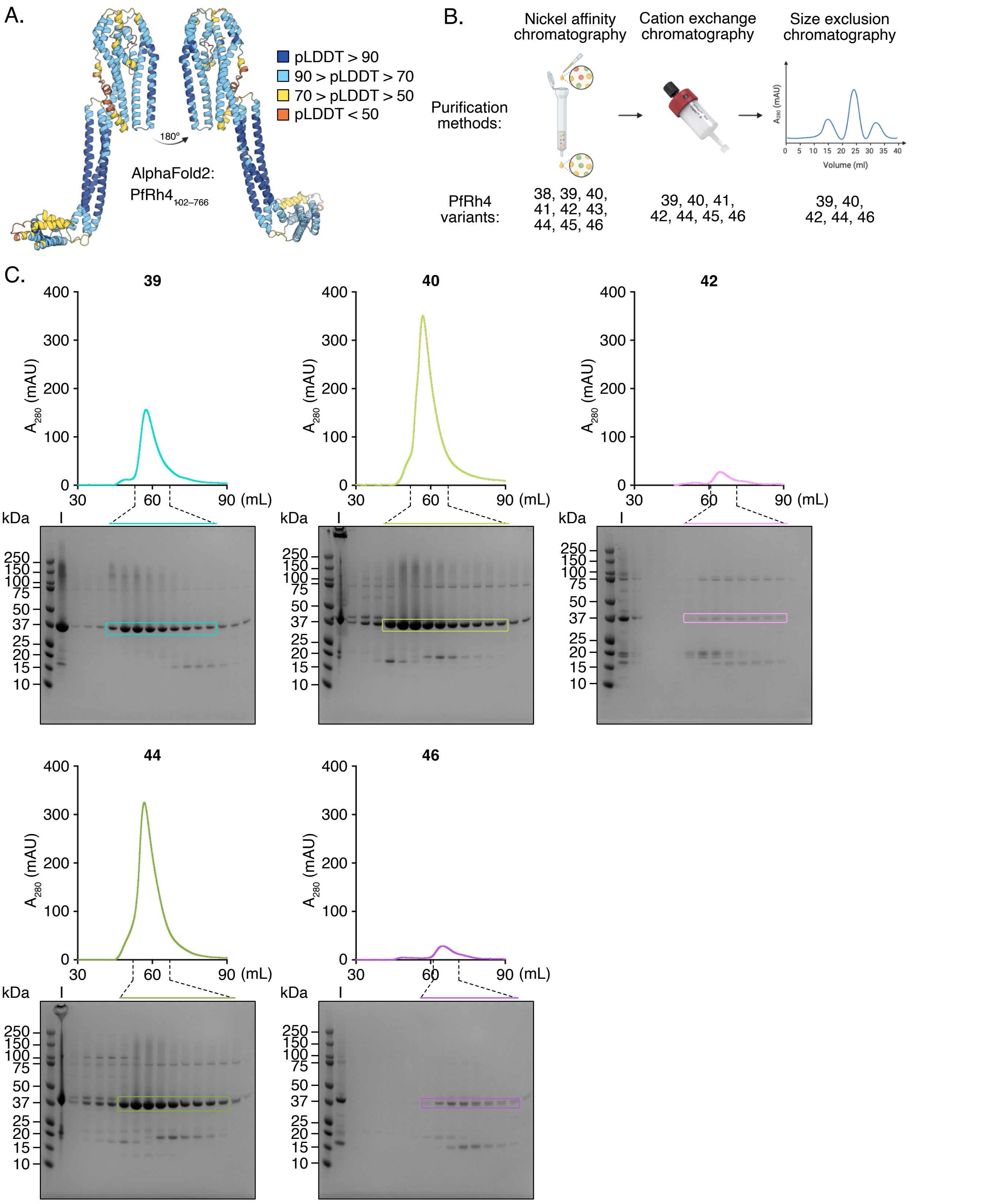

Figure S7. PfRh4 model and purification of stabilized PfRh4 variants. (A) Alphafold model of WT PfRh4$_{102\text{-}766.}$ Structure colored based on model confidence score (pLDDT) confidence with blue being high confidence and orange being low confidence. (B) Schematic of the three-step purification process for PfRh4 variants. Variants taken through each purification step are listed below each step. (C) SEC chromatogram and corresponding reduced SDS-PAGE gels of variants. Fractions that were pooled as the final purified protein are indicated by the dotted lines and corresponding box on the SDS-PAGE gel. I, input.

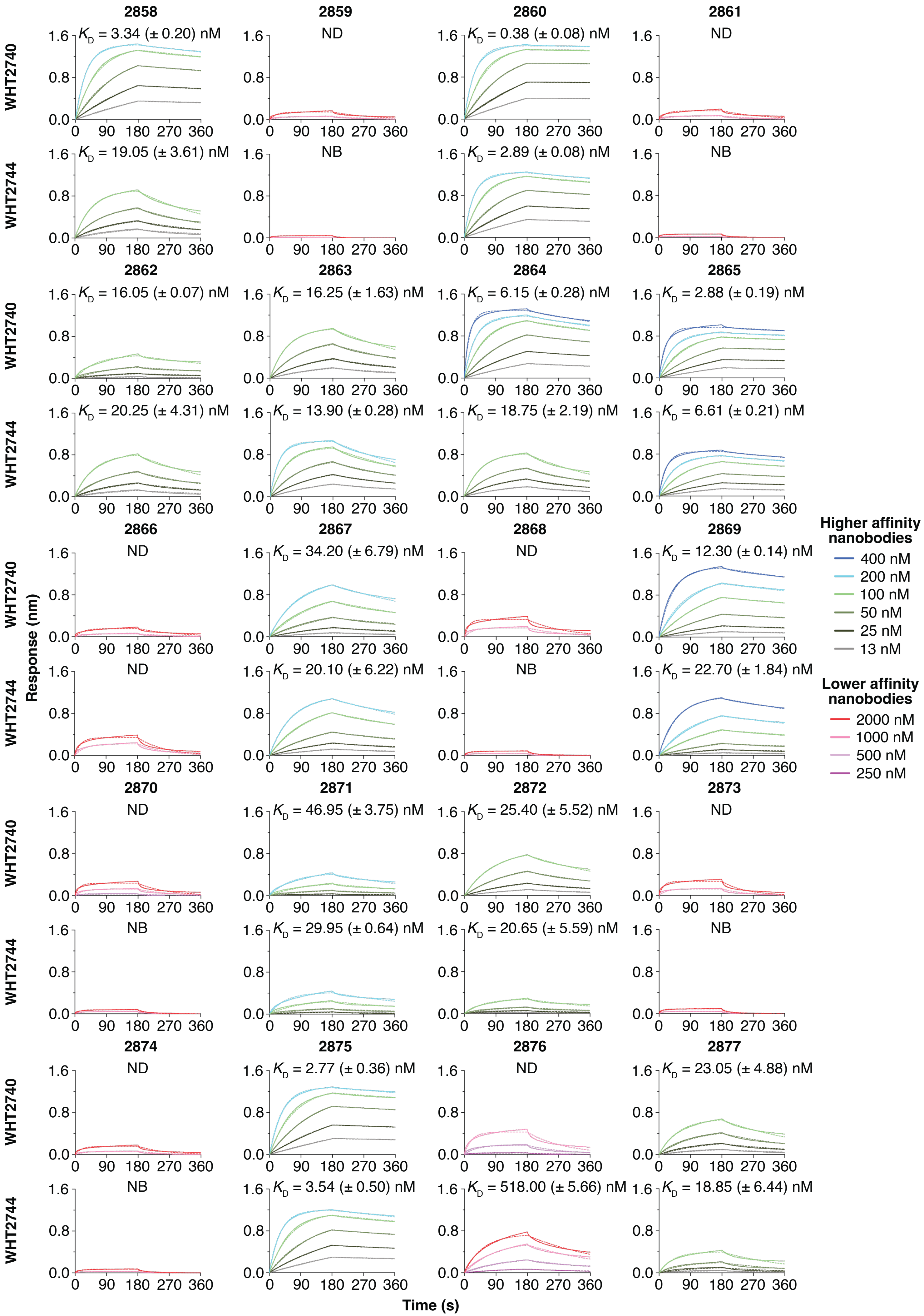

2858
WHT2740 K_D = 3.34 (± 0.20) nM
WHT2744 K_D = 19.05 (± 3.61) nM
2859
WHT2740 ND
WHT2744 NB
2860
WHT2740 K_D = 0.38 (± 0.08) nM
WHT2744 K_D = 2.89 (± 0.08) nM
2861
WHT2740 ND
WHT2744 NB
2862
WHT2740 K_D = 16.05 (± 0.07) nM
WHT2744 K_D = 20.25 (± 4.31) nM
2863
WHT2740 K_D = 16.25 (± 1.63) nM
WHT2744 K_D = 13.90 (± 0.28) nM
2864
WHT2740 K_D = 6.15 (± 0.28) nM
WHT2744 K_D = 18.75 (± 2.19) nM
2865
WHT2740 K_D = 2.88 (± 0.19) nM
WHT2744 K_D = 6.61 (± 0.21) nM
2866
WHT2740 ND
WHT2744 ND
2867
WHT2740 K_D = 34.20 (± 6.79) nM
WHT2744 K_D = 20.10 (± 6.22) nM
2868
WHT2740 ND
WHT2744 NB
2869
WHT2740 K_D = 12.30 (± 0.14) nM
WHT2744 K_D = 22.70 (± 1.84) nM
2870
WHT2740 ND
WHT2744 NB
2871
WHT2740 K_D = 46.95 (± 3.75) nM
WHT2744 K_D = 29.95 (± 0.64) nM
2872
WHT2740 K_D = 25.40 (± 5.52) nM
WHT2744 K_D = 20.65 (± 5.59) nM
2873
WHT2740 ND
WHT2744 NB
2874
WHT2740 ND
WHT2744 NB
2875
WHT2740 K_D = 2.77 (± 0.36) nM
WHT2744 K_D = 3.54 (± 0.50) nM
2876
WHT2740 ND
WHT2744 K_D = 518.00 (± 5.66) nM
2877
WHT2740 K_D = 23.05 (± 4.88) nM
WHT2744 K_D = 18.85 (± 6.44) nM
Response (nm)
Time (s)
0.0
0.8
1.6
0
90
180
270
360
Higher affinity nanobodies
400 nM
200 nM
100 nM
50 nM
25 nM
13 nM
Lower affinity nanobodies
2000 nM
1000 nM
500 nM
250 nM

Figure S8. BLI affinity curves of PfRh4 nanobodies binding to PfRh4 variants. BLI affinity measurements using a two-fold serial dilution of PfRh4 variants from 13 – 400 nM for higher affinity nanobodies and 250 – 2000 nM for lower affinity nanobodies binding to immobilized PfRh4 nanobodies. Measurements were plotted (solid line) and fitted to a 1:1 binding model (dashed line). Mean K_D values with standard deviations are indicated, and representative binding curves are shown from two independent experiments. NB, no binding; ND, not determined due to poor fitting of the measured data to the binding model.

Table S1: **Mutated residues between $PfRh4_{102–442}$ and variants**. Solvent accessible residues are coloured blue and are defined by having a relative surface accessible area (RASA) of 25% or greater.

	2739	2740	2741	2742	2743	2744	2745	2746
1	N111D	R112K	M114I	M114I	N1111D	M114I	M114I	M114I
2	R112L	M114I	D150E	C155M	M114I	I170L	C155M	C155M
3	M114I	D150E	C155M	I170L	L116I	F172L	I170L	I170L
4	Y115K	S157E	S157E	F172K	T154L	K203I	F172L	F172K
5	L116I	S168A	I170L	W186Y	C155M	Y217L	K203I	K203I
6	R140K	I170L	F172L	K203I	I170L	I240L	Y217L	Y217L
7	D150E	F172L	W186Y	E210I	F172L	S243T	L247F	C249F
8	T154L	W186Y	K203I	Y217L	K203I	C249F	C249F	V318A
9	C155M	K203I	M206L	H228R	I207L	I256L	Y252L	Y330F
10	S157Q	M206K	E210I	C249F	Y217L	V318N	V318C	E366T
11	N164Q	E210I	Y217L	V318A	I240L	Y330F	Y330F	
12	S168A	Y217L	H228R	Y330F	S243T	V337I	V337I	
13	K169N	H228R	L247F	E366T	C249F	E366T	E366T	
14	I170L	I240L	C249F	K395I	Y252W	Y367I	Y367I	
15	F172L	S243T	Y252L		I256L	T376L	T376L	
16	K173I	C249F	T266S		H312L			
17	W186Y	I256L	V318C		V318A			
18	E199L	T266D	Y330F		S319A			
19	K203I	H312K	V337I		I326L			
20	M206K	V318N	F354L		E327L			
21	I207L	Y330F	E366T		Y330F			
22	E210I	V337I	Y367I		V337I			
23	Y217L	F354L	T376L		K339L			
24	K220E	E366T	K395L		Y351F			
25	H228R	Y367I			E366T			
26	I240L	T376L			Y367I			
27	S243T	K395I			I371L			
28	C249F				T376L			
29	Y252W							
30	I256L							
31	T266D							
32	H312L							
33	V318A							
34	S319A							
35	I326L							
36	E327L							
37	Y330F							
38	V337I							
39	K339L							
40	Y351F							
41	F354L							
42	E366T							
43	Y367I							
44	I371L							
45	T376L							
46	S386E							
47	S390T							
48	K395I							
49	Q409K							
