# Supplementary material for: Nanobodies against Plasmodium adhesins that block receptor engagement and malaria parasite invasion
Source: Biochem J. 2026 Jul 9;483(8):1473–88. doi: 10.1042/BCJ20260354 (PMC13358728; doi:10.1042/BCJ20260354)
Supplement: Supplementary Dataset S1-S4 [file BCJ-2026-0354_supp1.zip › Supplementary-Dataset.docx]

**Supplementary data**

**Data S1. Data relating to nanobody characterization performed in Figure 1**. Table of nanobody CDR3 sequences, full-length (FL) sequences, their corresponding counts per million (CPM) from NGS, enrichment from Sanger sequencing and determined binding kinetics and affinity data. Data are from two independent biolayer interferometry experiments and shown are the mean ± SD values for affinity (*K*_D_), association rate (*k*_a_) and dissociation rate (*k*_d_). Chi-squared (X^2^) and R-squared (R^2^) are statistical values indicating quality of fit between experimental data and theoretical model.ND, not determined; NB, no binding; NE, no expression.

**Data S2. Single binding data for PvRBP2b and PfRh4 nanobodies against the N-terminal conserved scaffold**. (A) Binding response of PvRBP2b nanobodies to PvRBP2b_161–1454_, PvRBP2b_169–470_ and PvRBP2b_169–470_ stabilized design (2483) from one experiment as measured by biolayer interferometry. (B) Binding response of PfRh4_102–766_ mouse monoclonal antibodies to PfRh4_102–766_ and variants from two independent experiments with mean ± SD values shown.

**Data S3**. Replicate IC_50_ values for PfRh5 nanobodies, PfRh5 nanobody-Fcs and PfRh4 nanobodies.

**Data S4**. Accessible surface area, relative surface area and relative accessible surface area for residues in PfRh4 variant designs.
